# Case Report: Sonographic Detection of Chorionic Hemangiomas in Sturge-Weber Syndrome

**DOI:** 10.5070/M5.52314

**Published:** 2026-10-31

**Authors:** Irina Hariri, Mohammed Haq, Michael McGee

**Affiliations:** * Indiana University School of Medicine, Department of Emergency Medicine, Indianapolis, IN; ^ Indiana University School of Medicine, Department of Emergency Medicine, Department of Pediatrics, Indianapolis, IN

## Abstract

Point-of-care ultrasound (POCUS) is used frequently in the emergency department (ED) as a non-invasive diagnostic tool. Ocular POCUS is utilized in patients with vision changes to detect pathologies including retinal detachment, vitreous hemorrhage and central retinal artery occlusion. This case demonstrates the capability of POCUS to detect choroidal hemangiomas, a pathology strongly associated with Sturge-Weber syndrome (SWS). A 31-year-old male with no significant medical history presented with two years of progressive vision changes and headaches. A large right-sided facial birthmark was noted, though the patient had no prior diagnosis. Examination revealed right eye blindness and an afferent pupillary defect. Ocular POCUS revealed retinal detachment and choroidal thickening, which were interpreted as choroidal hemangiomas. Findings were corroborated by MRI. Together with input from our ophthalmology and neurology specialists, a diagnosis of SWS was made. This case demonstrates the utility of POCUS in the rapid identification of choroidal hemangiomas in an adult patient, facilitating an expedited diagnosis of SWS. Because there is no cure for SWS, early diagnosis and management by a multidisciplinary team, including ophthalmology and neurology, is essential to limit the progression of the disease and mitigate its effects.

**Topics:**

Ocular, hemangiomas, chorion, Sturge-Weber, POCUS.

**Figure:**
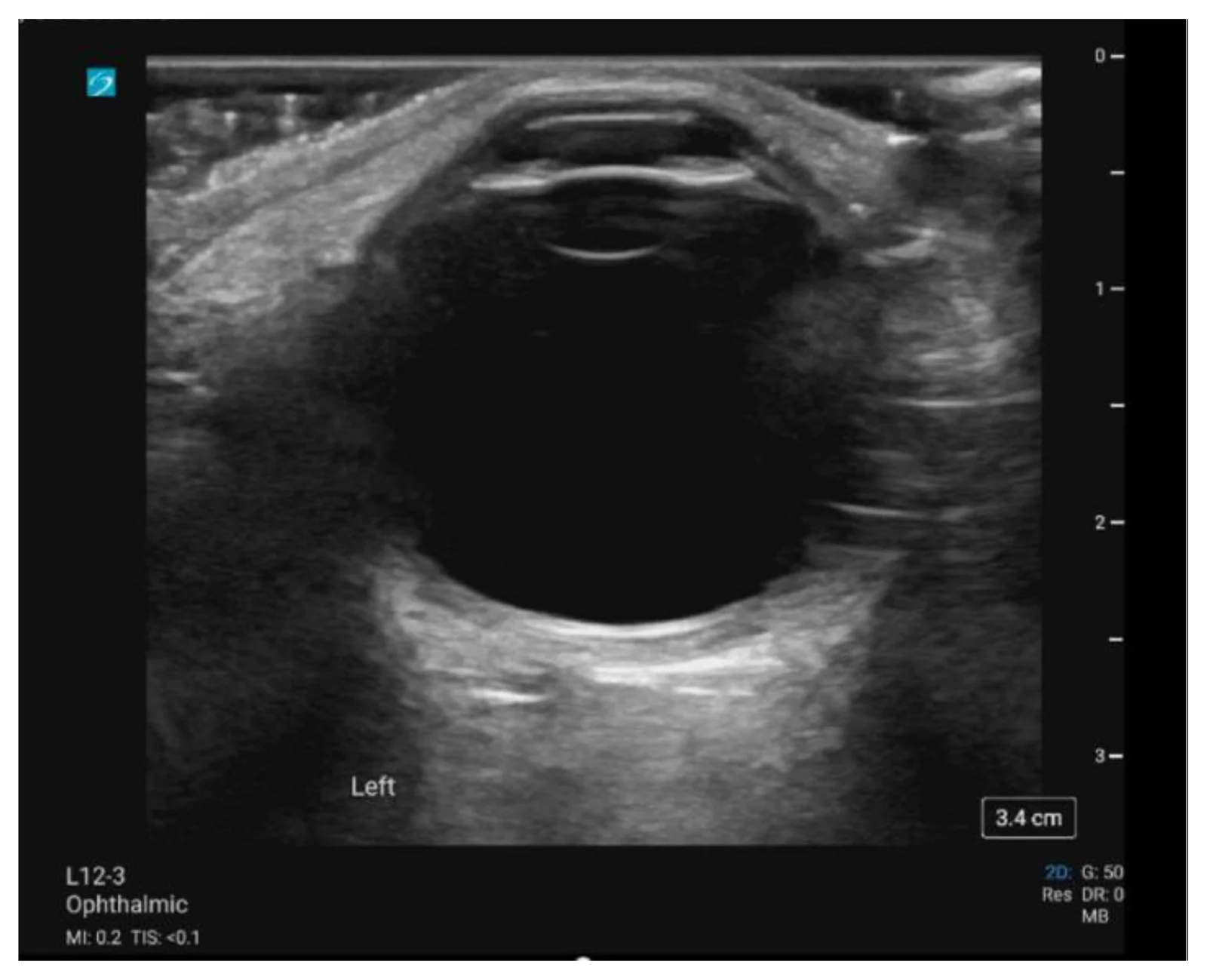


**Figure:**
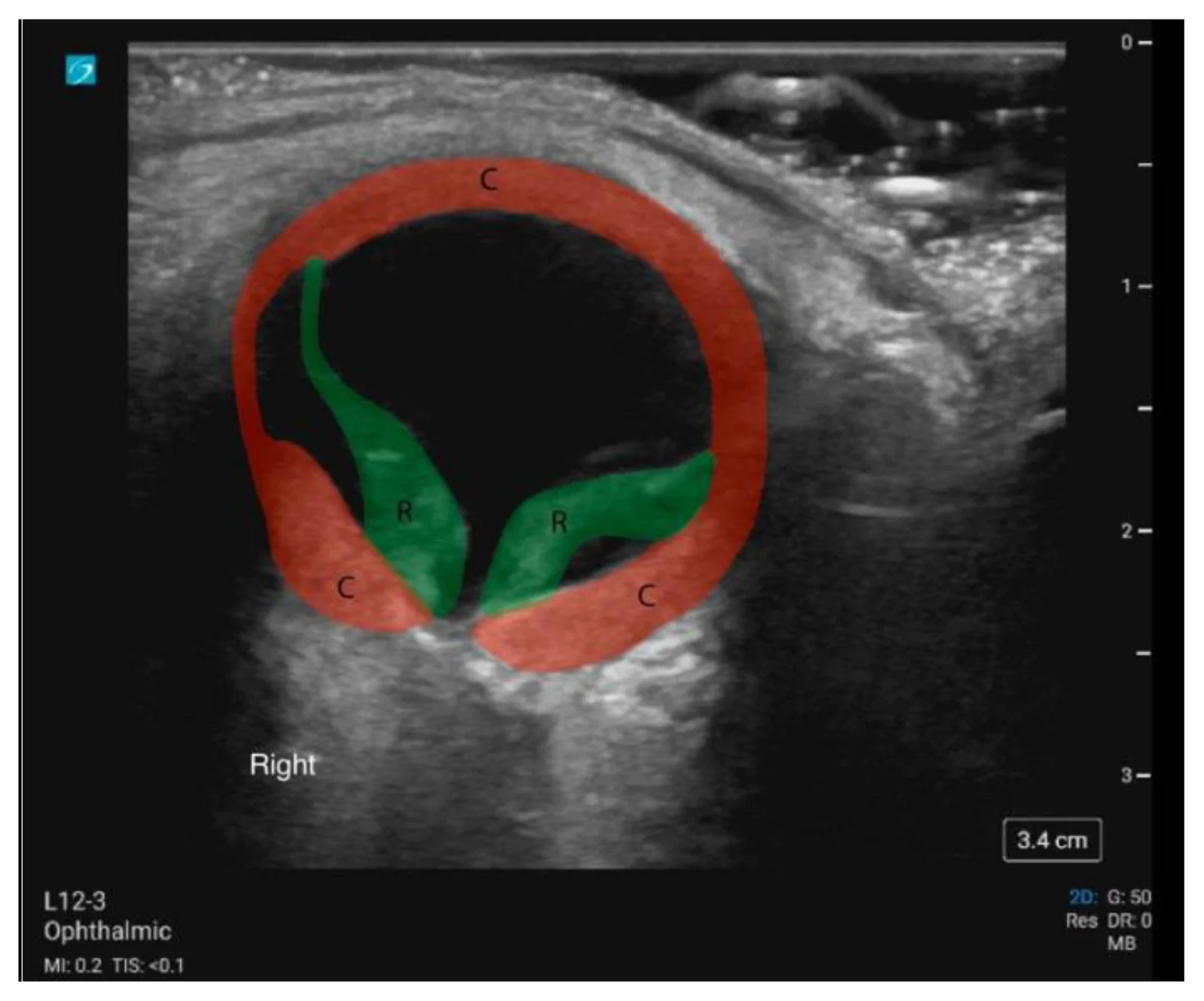


## Brief introduction

Sturge-Weber syndrome (SWS) is a rare congenital neurocutaneous disorder. Various capillary malformations result in port-wine colored birthmark on the face, glaucoma, and neurological manifestations which include seizures and leptomeningeal angiomatosis. It occurs as a sporadic mutation of the GNAQ gene with an estimated frequency between 1/20,000 and 1/50,000 live births.^1^ It affects males and females equally and has no association with race. The facial birthmark and ocular/leptomeningeal capillary defects are usually ipsilateral to each other. Seizures, present in 75% of patients, classically become refractory over time.^2^ Cognitive deterioration is common secondary to a combination of recurrent seizures, venous engorgement leading to increased cerebral pressure, intracranial calcifications, and ischemic events.

Many cases of SWS are suspected in infancy due to the presence of the characteristic facial birthmark. Most diagnoses are made before the age of two, with up to 90% of patients experiencing seizures within the first year of life. 1,3 Rarely, individuals may not exhibit any symptoms in childhood. There are documented cases of diagnoses of SWS in adults, though typically involving patients who presented with symptoms in childhood but either remained undiagnosed or whose disease was undetectable at the time. One case study describes a woman who developed symptoms of SWS in her sixties.^4^

## Presenting concerns and clinical findings

A 31-year-old male with no known past medical history except a large right-sided facial birthmark presented with two years of progressively worsening right-sided vision loss and headaches. He had the facial birthmark for as long as he could remember but never had any associated diagnosis. Beginning two years ago, without any inciting factors, he noted his right eye became progressively blurrier, and one year ago he lost sight entirely. He also reported sharp, intermittent pain in that eye. Over the same period of time, he noted right-sided, intermittent, throbbing headaches with associated nausea and vomiting that lasted hours or days. He denied focal neurologic deficits, fevers, chills, weight loss, or night sweats. He denied history of seizures.

The physical examination was notable for a large, purple-hued birthmark extending from his right forehead down to his right mandible, involving the upper and lower eyelid margins. His ophthalmic examination of the right eye was remarkable for a complete loss of light perception and an afferent pupillary defect. His left eye visual acuity was unaffected, and the intraocular pressures were within normal limits bilaterally. The remaining neurologic examination was without focal findings. The patient’s laboratory workup showed mild leukocytosis, while other tests, including inflammatory markers, were unremarkable.

## Significant findings

Ocular point-of-care ultrasound (POCUS) was performed using a linear 12–3 MHz probe on a Sonosite PX system. Examination of the symptomatic right eye revealed a complete retinal detachment. The detached retina appeared thickened, consistent with chronic detachment. Deep to the retina, the choroid was markedly thick with the widest portion observed in the posterior wall of the globe near the optic nerve.

Sonographically, the choroid was isoechoic to the retina in the near field, hyperechoic to the retina in the far field (secondary to posterior acoustic enhancement) and homogenous. In the colorized image of the right eye, the red color denotes the choroid, labeled C, and the green color denotes the retina, labeled R. The left eye was also examined, shown in the last case image, and was grossly normal. No color doppler imaging was utilized for either eye. Optic nerve sheath diameter (ONSD) was 0.54 cm bilaterally.

## Patient course

Given the clinical suspicion of a neurocutaneous disorder such as SWS, our team consulted both ophthalmology and neurology. Magnetic resonance imaging of the brain revealed enhancement of the choroid plexus in the right lateral ventricle and angiomatosis of the right calvarium. The MRI did not show other intracranial findings strongly associated with severe cases of SWS, such as cortical or subcortical calcifications. Orbital MRI confirmed choroidal hypertrophy and retinal detachment in the right eye. Based on the combination of the patient’s facial birthmark, choroidal hemangiomas and imaging results, a presumptive diagnosis of SWS was made the same day.

The patient was discharged from the emergency department with plans for outpatient follow-up with neurology and ophthalmology. While treatments for choroidal hemangiomas are available in the earlier stages, the vision in the patient’s right eye is unlikely to improve following the retinal detachment. Therefore, the primary focus of management will be to preserve vision in the left eye. The patient was initiated on amitriptyline to reduce headache frequency and vitamin D supplementation (a common deficiency observed in individuals with SWS). Prophylactic anti-epileptic medications and acetylsalicylic acid (ASA) were not prescribed during this visit given the absence of seizure activity and the lack of significant intracranial involvement on imaging. Additional neurological evaluation, including neurocognitive testing, is planned for the outpatient setting.

## Discussion

Sturge-Weber syndrome is associated with a high prevalence of ophthalmologic involvement, with 30–70% of those individuals developing glaucoma.^4^ The second most common ophthalmologic manifestations are diffuse choroidal hemangiomas, benign vascular tumors, affecting up to 40–50% of patients.^5^ These tumors have a range of presentations clinically but include decreased visual acuity, refractive errors, and retinal detachment. Choroidal hemangiomas are the likely etiology of our patient’s retinal detachment that ultimately caused his vision loss. Other potential abnormalities include discoloration of the iris, proptosis, and tortuous vessels and hemangiomas of the conjunctiva and episclera.

Ocular POCUS is one of the many tools currently employed by ophthalmologists to monitor ocular involvement in SWS patients. In this case, the use of POCUS directed us to seek specialty consultation and advanced imaging, which ultimately facilitated a timely diagnosis in the ED. However, it is important to note that a diagnosis of SWS cannot be made solely based on the presence of choroidal hemangiomas. The definitive diagnosis requires a comprehensive evaluation of clinical symptoms in conjunction with MRI findings.^1^

To our knowledge, there is no published research comparing the sensitivity of ocular POCUS to MRI for detecting choroidal hemangiomas specifically. In a study of 12 eyes of patients with Primary Choroidal Malignant Melanoma, Romani, et al., found ultrasound to be 100% sensitive, 92% specific compared to MRI which had a sensitivity and specificity of 100% and 83%, respectively.^6^ One limitation of POCUS, as compared to MRI, is the two-dimensional nature, which may result in missed focal abnormalities if the entire ocular globe is not carefully assessed during the examination.

Currently, there is no definitive cure for SWS. The management strategy primarily focuses on supportive care aimed at preventing or minimizing serious complications, such as seizures, vision loss, and stroke-like events. While research into managing the pre-symptomatic phase of the condition is ongoing, there is limited evidence supporting the efficacy of prophylactic ophthalmologic interventions.^7^ Several treatment strategies are available for managing ocular complications once they occur.,^7^,^8^ Given the complexity of SWS, a multidisciplinary approach is essential for patient care. Regular screenings are crucial to identify and address ocular manifestations early.^1^,^9^

## Supplementary Information






